# Supplementary material for: In Situ Modification of Multi-Walled Carbon Nanotubes with Polythiophene-Based Conjugated Polymer for Information Storage
Source: Materials (Basel). 2023 Jan 18;16(3):908. doi: 10.3390/ma16030908 (PMC9918207; doi:10.3390/ma16030908)
Supplement: Supplementary file 1 [file materials-16-00908-s001.zip › materials-2069967-supplementary.pdf]

*materials*

*Supplementary Materials*

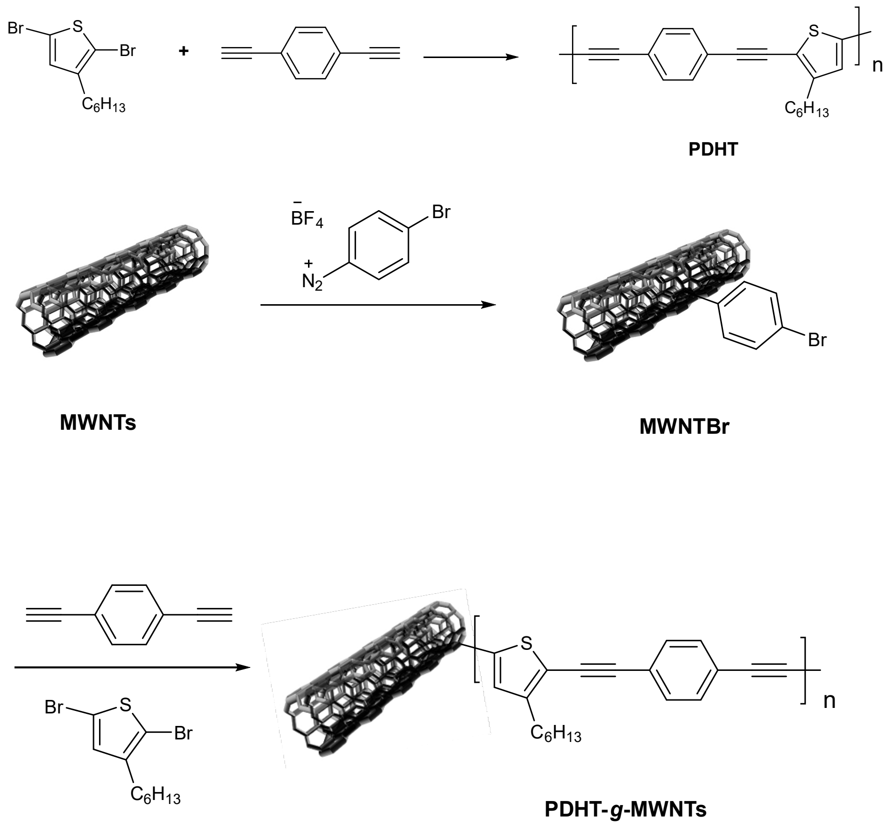


**Scheme S1.** Synthetic routes of PDHT and PDHT-*g*-MWNTs.

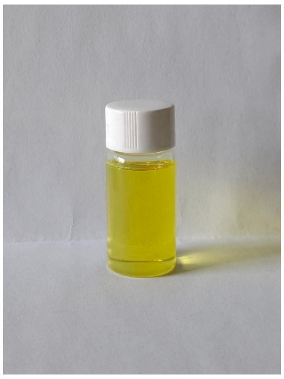

**Figure S1.** The digital photo of PDHT dispersed in NMP: the sample dispersed immediately after sonication. Concentration of the sample was 5 mg/mL.

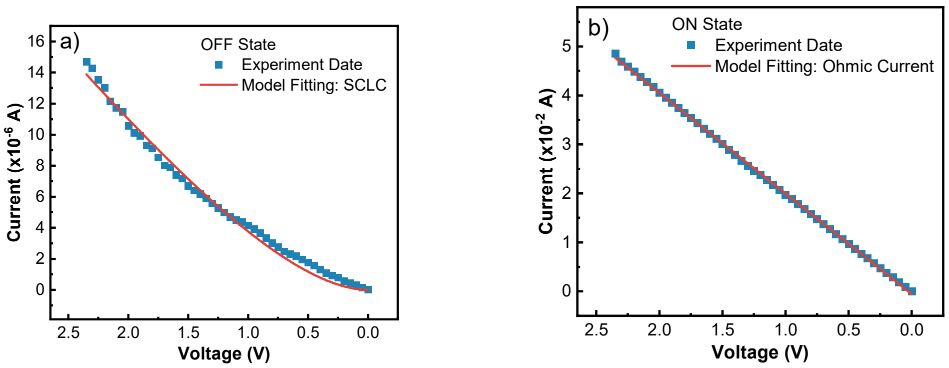


**Figure S2.** Experimental data and fitted *I-V* characteristics for the as-fabricated memory device in the OFF state (**a**) and ON state (**b**).

Without an input voltage, the electrons in the system were stable, implying that the as-fabricated Al/PDHT-*g*-MWNTs/ITO device was in the OFF state. The OFF state current was fitted by a space-charge-limited current (SCLC) model (**Figure S2a**):

$$J = A\mu\varepsilon\varepsilon_0 \frac{V^2}{d^3} \exp\{\frac{0.891}{kT}(\frac{q^3V}{\pi\varepsilon\varepsilon_0 d})^{1/2}\} \quad (1)$$

where $J$, $A$, μ, $\varepsilon\varepsilon_0$, $d$, $V$, $k$, $T$, and $q$ are the current density, positive constant, the mobility of charge carriers, the absolute permittivity of the complex, the thickness of the film, voltage, the Boltzmann constant, the ambient temperature, and the absolute value of the unit electronic charge ($1.6 \times 10^{-19}$ C), respectively. The ON state current was also fitted by an Ohmic current model (**Figure S2b**)

$$J = B\frac{qnv}{d} \quad (2)$$

where $B$ is a positive constant and $n$ is the charge carrier density. The electron delocalization in MWNTs stabilized the conductive CT state, resulting in the nonvolatile nature of the ON state.

**Disclaimer/Publisher's Note:** The statements, opinions and data contained in all publications are solely those of the individual author(s) and contributor(s) and not of MDPI and/or the editor(s). MDPI and/or the editor(s) disclaim responsibility for any injury to people or property resulting from any ideas, methods, instructions or products referred to in the content.
